# Modifiable Factors of Health-Related Quality of Life Among LGBTQ Older Adults Living With Cognitive Impairment

**DOI:** 10.1093/geroni/igab046.427

**Published:** 2021-12-17

**Authors:** Hyun-Jun Kim, Karen Fredriksen Goldsen

**Affiliations:** University of Washington, Seattle, Washington, United States

## Abstract

Cognitive impairment can lead to significant decline in health-related quality of life (HRQOL) in later life especially among those who are socially marginalized. While Lesbian, gay, bisexual, transgender, and queer (LGBTQ) older adults are documented to be at heightened risks of cognitive impairment, they may face unique challenges due to discrimination, social isolation, and other LGBTQ-related risks. This study examined factors associated with psychological and physical HRQOL among LGBTQ adults aged 50 and older analyzing a sub-set of longitudinal data (N = 646) from National Health, Aging, and Sexuality/Gender Study: Aging with Pride. Lifetime LGBTQ discrimination and victimization and insufficient food intake were negatively, and physical and leisure activities were positively associated with both HRQOL dimensions. Community engagement, social support, and social activities were positively associated with psychological HRQOL. Culturally responsive interventions addressing these modifiable factors are needed to improve HRQOL of this socially marginalized but resilient population.

